# Robust Interferometry for Testing Thermal Expansion of Dual-Material Lattices

**DOI:** 10.3390/ma13020313

**Published:** 2020-01-09

**Authors:** Weipeng Luo, Shuai Xue, Cun Zhao, Meng Zhang, Guoxi Li

**Affiliations:** College of Intelligent Science, National University of Defense Technology, Changsha 410073, China

**Keywords:** dual-material lattices, interferometry, measurement, thermal analysis, thermal expansion

## Abstract

Dual-material lattices with tailorable coefficients of thermal expansion have been applied to a wide range of modern engineering systems. As supporting techniques for fabricating dual-material lattices with given coefficients of thermal expansion, the current existing methods for measuring the coefficient of thermal expansion have limited anti-interference ability. They ignore the measuring error caused by micro-displacement between the measurement sensor and the test sample. In this paper, we report a robust interferometric test method which can eliminate the measurement error caused by micro-displacement between the measurement sensor and the test sample. In the presented method, two parallel plane lenses are utilized to avoid the measurement error caused by translation, and the right lens is utilized as an angle detector to eliminate the measurement error caused by rotation. A robust interferometric testing setup was established using a distance measuring set and two plane lenses. The experiment results indicated that the method can avoid the measurement error induced by translation and has the potential to eliminate the measurement error induced by rotation using the rotational angle. This method can improve the anti-interference ability and accuracy by eliminating the measurement error. It is especially useful for high-precision thermal expansion measurement of dual-material lattices.

## 1. Introduction

Dual-material lattices with tailorable coefficients of thermal expansion (CTE) have been widely used in many applications [1,2,3]. Various dual-material lattices have been proposed to obtain tailorable CTEs [4,5,6,7,8]. In order to guide their design and machining, the equivalent CTEs of the dual-material lattices must be accurately measured ahead. Usually, the CTE measuring process takes a long time due to the slow heating. During this long process, vibrations from the environment and the thermal deformation of the measuring device will cause micro-displacement between the measurement sensor and the test sample. The micro-displacement will generate unacceptable measurement error for high-precision measurement. Hence, developing a robust measurement method for testing the CTEs of dual-material lattices is meaningful to enhance measurement accuracy. However, the existing thermal expansion measurement CTE technologies are sensitive to the micro-displacement between the measurement sensor and the test sample.

Various techniques are available to measure the CTE of materials, including strain gauge technique [9,10], capacitance method [11,12], interferometric technique [13,14], and digital image correlation (DIC) method [1,15,16]. Among these, laser interferometric techniques and the DIC method are the most used to measure the CTEs of dual-material lattices. The laser interferometric technique uses interference fringe variation to measure thermal deformation with high accuracy. However, the laser interferometric fringe pattern is very sensitive to the vibration of the environment, and this method cannot eliminate the measurement error caused by the micro-displacement [13,14]. We measured the CTE of a dual-material lattice with negative thermal expansion using laser interferometry [17], but the measurement error was large, and we did not consider the measurement error caused by the micro-displacement. Digital image correlation provides a full-field thermal deformation by comparing the images captured before and after deformation [18]. It uses a high-resolution camera to capture the digital images of the test sample. This method can eliminate part of the measurement error caused by the micro-displacement via data processing. However, this method is sensitive to temperature variation, air turbulence, and out-of-plane displacement of the sample [19,20]. Therefore, developing a robust measurement method for testing the CTEs of dual-material lattices which can eliminate the measurement error caused by micro-displacement is still challenging and has never been reported (according to the authors’ knowledge).

In this paper, we report on a robust laser interferometric measurement method for dual-material lattices to overcome the measurement error caused by micro-displacement. The presented method has high anti-interference capability by using a distance measuring system. The distance measuring system consists of a distance measuring set and two parallel plane lenses. The two parallel lenses can avoid the measurement error caused by the translational component of the micro-displacement. The right lens, working as a micro-rotation angle indicator, can measure the rotational angle of the micro-displacement. The measurement error caused by the rotational component of the micro-displacement is eliminated with the rotational angle. With this method, the measurement error caused by micro-displacement can be eliminated completely.

## 2. Thermal Expansion Measurement System

### 2.1. Principle of the Measurement System

**Figure 1:**
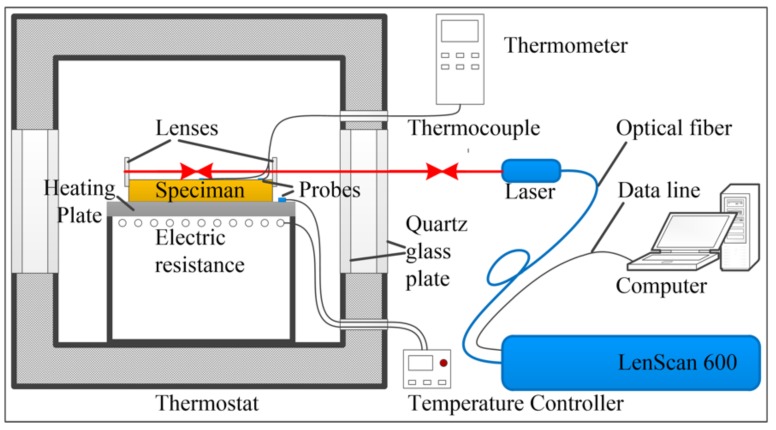
Schematic diagram of the measurement system. It consists of a temperature control system and distance measurement system.

The schematic diagram of the experimental system for CTE measurement is shown in Figure 1. It includes a temperature control system and distance measurement system. The temperature control system consists of a thermostat, an electric heating plate, a temperature controller, and a thermocouple thermometer. The thermostat is used to provide uniform ambient temperatures for the sample to reduce temperature inhomogeneity. The electric heating plate is heated by the electric resistance. It uses the temperature controller to realize the temperature control. This electric heating plate is used for rapid heating of the sample. The thermocouple thermometer has two thermocouple probes to monitor the temperature at different locations of the sample. It can indicate whether the temperature of the test sample is uniform.

The distance measuring system is composed of a distance measuring set and two plane optical lenses. The two lenses are mounted on both ends of the sample to reflect the laser beam. The right lens, near the laser, is used to measure the rotational angle between the sample and the laser beam.

### 2.2. Establishment of the Experimental System

**Figure 2:**
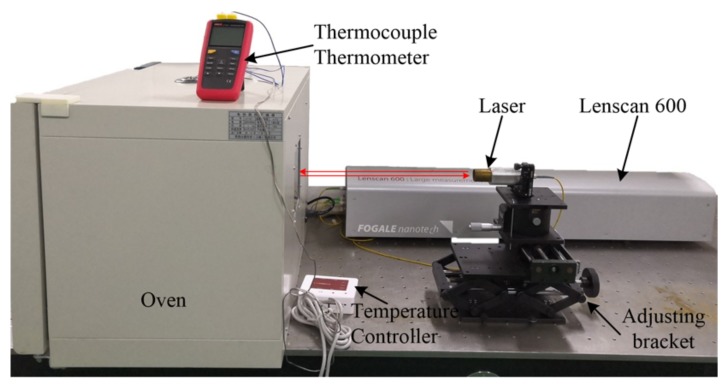
Actual experimental setup. The oven, thermocouple thermometer heating plate, and temperature controller form the heating system. The Lenscan 600 (Nimes, France) was used to measure the distance.

The actual experimental setup for the CTE measurement is shown in Figure 2. In the actual experimental setup, an oven (Lichen-101BS, Shanghai, China) with temperature control was used as the thermostat. The temperature controller (HS-618F, Shanghai, China), with an accuracy of ±1 °C, was used to control the temperature of the heating plate. It allows non-contact temperature settings via an infrared remote controller. The thermocouple thermometer (UT320, Dongguan, China), with two thermocouple probes, was used to monitor the temperature at different locations of the sample.

The distance measuring set (LS600, Nimes, France), with an absolute accuracy of ±1 μm, was used to measure the thermal deformation of the sample. It can measure the length of the air gap between the two lenses and the thickness of the right lens based on low coherence interferometry [21]. The two plane lenses (N-BK7, Shanghai, China) were fixed on the sample by two mounting brackets. Each mounting bracket had three angle adjusting screws. Thus, the angles of the lenses could be adjusted to allow the reflected laser to coincide with the incident laser beam.

### 2.3. Measurement Steps

**Figure 3:**
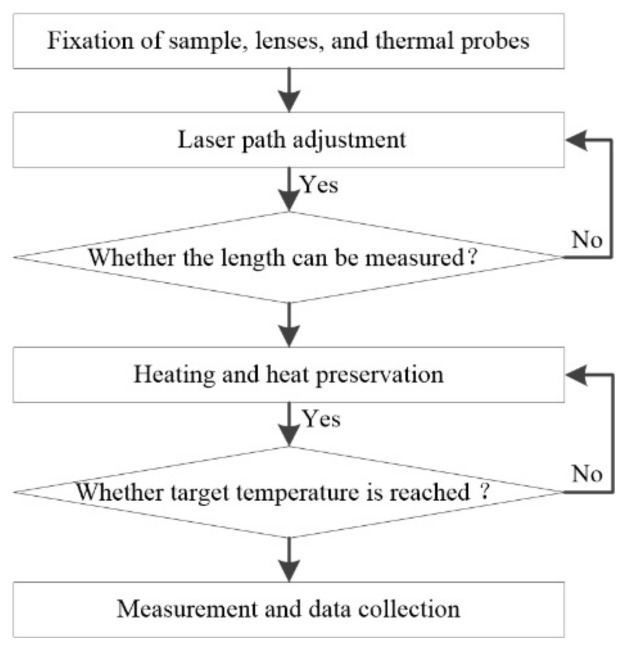
Flowchart of the steps for coefficient of thermal expansion (CTE) measurement.

The following steps were the experimental measurement procedures as shown in Figure 3.

#### 2.3.1. Fixation of the Sample, Lenses, and Thermocouple Probes

The sample was fixed on the surface of the heating plate. The two thermocouple probes were fixed at different positions on the sample by high-temperature rubberized fabric. Then, the two lenses were fixed on the sample using the two mounting brackets.

#### 2.3.2. Laser Path Adjustment

First, the height of the adjusting bracket was adjusted to ensure the center height of the laser probe was consistent with the center height of the two lenses. Second, the pitch angle and yaw angle of the laser probe were adjusted to ensure that the laser beam could pass through the center of the two lenses. Third, the angle adjusting screws of the two lenses were adjusted to reflect the light point to coincide with the incident point. Each lens had to be adjusted independently. Then, the length of the air gap between the two lenses was measured to determine whether the measurement quality was acceptable; if it was not, the laser path was readjusted.

#### 2.3.3. Heating and Heat Preservation

First, the temperature of the thermostat and the heating plate were set at the target temperatures and the sample was heated. When the thermostat and hot plate both reached their target temperatures, they were kept warm for approximately half an hour. Second, the thermocouple thermometer was used to monitor the temperature at different positions of the sample. The readings of the two thermocouple probes were observed until the difference between the two readings was less than 0.5 °C.

#### 2.3.4. Measurement and Data Collection

The sample was measured five times at each temperature point and the results recorded. Then, the temperature setting was changed, and Section 2.3.3 and Section 2.3.4 were repeated until the measurements were completed.

## 3. Measurement Error Analysis

**Figure 4:**
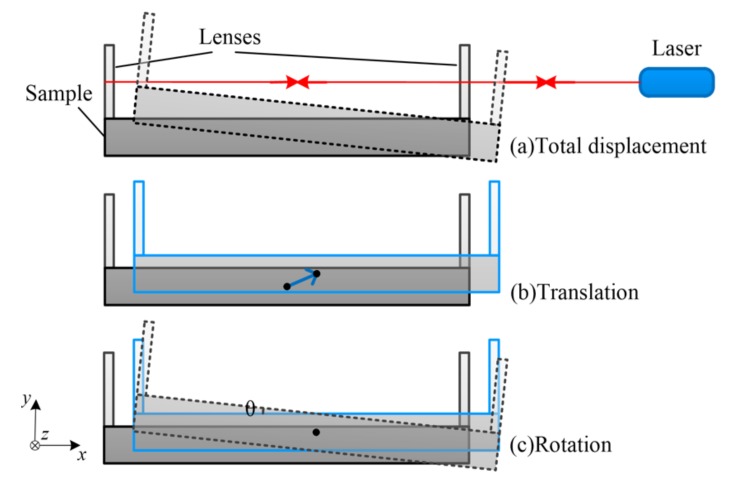
Schematic diagram of the micro-displacement decomposition. (**a**) Total displacement. (**b**) The translational component. (**c**) The rotational component.

During the heating process, in order to guarantee the uniformity and accuracy of the sample temperature, the sample was heated slowly. Thus, the whole measurement process needed a long time. Over such a long period of time, the vibration of the environment and the thermal deformation of the measuring system caused uncertain micro-displacement between the sample and the measurement sensor. This micro-displacement can be decomposed into a translational component and a rotational component as shown in Figure 4. First, we assumed that these plane lenses were rigidly connected to the sample: the micro-displacement of the sample and the micro-displacement of the lenses were the same. Second, we assumed that the reflected laser coincided with the incident laser in the initial conditions: the initial angle between the sample and the laser was zero. Third, we assumed that the sample did not warp during the measurements. The following analyzes the measurement errors caused by these two components and how to eliminate these errors.

During the measurement process, the temperature dependence of the refractive index must be considered. The distance measuring set measures the optical path based on a linear optical encoder [21]. Then, the actual air gap and thickness of the lens are calculated by the measurement software automatically. By setting the refractive index of each material in the initial measurement model, the measuring software can obtain the actual distance or thickness through calculation. However, in the process of thermal expansion measurement, the refractive indexes of the materials change with the temperature increase. This will lead to an inaccurate result. In order to improve the measurement accuracy, the influence of temperature on the refractive index should be considered.

### 3.1. Influence of the Translational Component

**Figure 5:**
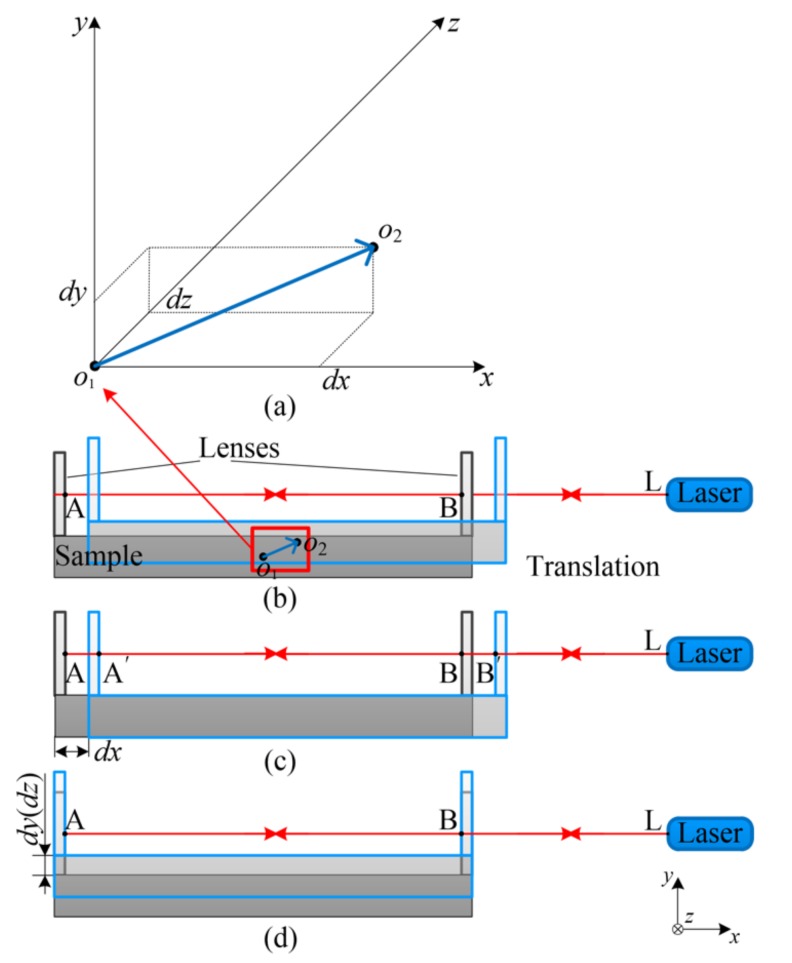
Schematic diagram of the translation displacement decomposition. (**a**) Translation displacement decomposition along the axis. (**b**) Total translation displacement. (**c**) The *x* component. (**d**) The *y* and *z* component.

The translational component is one of the sources of measurement errors. In this presented CTE measurement system, it measures the CTE by measuring the optical path of the air gap between the two lenses. The optical path of the air gap was obtained by the difference of the optical paths between the two lenses and the laser source as shown in Figure 5b. It can be expressed as:

(1) $$D_{AB}=D_{LA}-D_{LB}$$

In Equation (1), *D_AB_* is the optical path of the air gap, *D_LA_* is the optical path of the left lens and the laser source, and *D_LB_* is the optical path between of the right lens and the laser source. As shown in Figure 5b, the translational component can cause changes in the optical path between the lenses and the laser source.

The translational component can be decomposed along the axis into three components: *dx*, *dy*, and *dz* as shown in Figure 5a. Through the laser path adjustment, the lens’ surfaces are perpendicular to the laser beam. The two lenses are parallel to each other. Thus, *dy* and *dz* will not change distances between the lenses and the laser source as shown in Figure 5d. Therefore, *dy* and *dz* will not change the optical path of the air gap. When the sample moves along the *x*-axis, as shown in Figure 5c, the optical path of *D_LA_* and *D_LB_* change to *D_LA’_* and *D_LB’_*. However, the lengths of *D_LA_* and *D_LB_* decrease by the same value, *dx*. Thus:

(2) $$\frac{\partial D_{AB}}{\partial x}=0$$

In summary, the three translational components (i.e., *dx*, *dy*, and *dz*) will not change the optical path of the air gap. The translational component of the micro-displacement will not generate measurement errors for the CTE measurement. Thus, the measurement errors caused by the translational component can be avoid by the two parallel lenses.

### 3.2. Measurement Error Analysis and Elimination of Rotational Component

#### 3.2.1. Influence of Rotational Component

**Figure 6:**
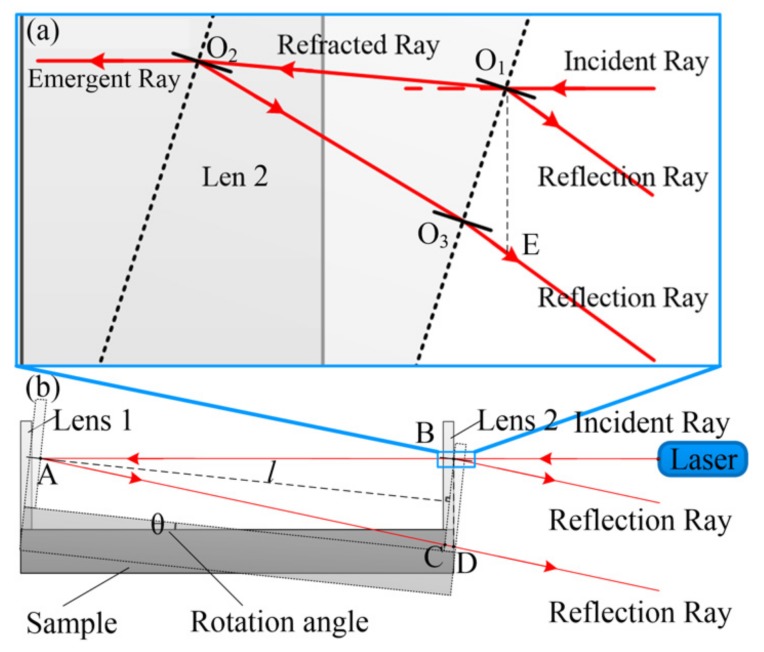
Schematic diagram of the laser transmission after the rotation. (**a**) The laser transmission at the right lens. (**b**) The laser transmission of the measurement system after rotation.

The rotational component is another source of measurement error. An excessive rotation angle will prevent the laser receiver from receiving reflected light. It will lead to measurement failure. The rotational component can be decomposed along the axis into three components in cartesian coordinates: around the *x*-axis, around the *y*-axis, and around the *z*-axis. The rotational component around the *x*-axis only makes the sample rotate around the laser beam. It does not cause extra optical path changes. Thus, it will not make measurement errors. Considering the rotational component around the *y*-axis and the rotational component around the *z*-axis, each component will cause the angle change between the sample and the laser beam. The situation will be more complex when the two components both occur. In order to simplify the calculation, the two components can be described in the polar coordinates. The total rotational component has just one direction and one angle to the laser beam. The direction of the total rotational component is circular symmetric. Thus, we can simplify the complicated situation into rotation around only one transverse axis. Take the rotation around the *y*-axis as an example for a small rotation angle; the incident and reflected rays no longer coincide as shown in Figure 6.

When the sample rotates for angle *θ*, the lenses are no longer perpendicular to the laser beam as shown in Figure 6b. According to the laws of reflection and refraction, the reflected rays will be deflected, and transmitted light will be refracted. Then, the optical path of the air gap changes. It can be expressed as:

(3) $$D_{gap}=\frac{D_{AB}+D_{AC}+D_{CD}}{2}=n_{1}l/\cos\theta +n_{1}l\sin^{2}\theta /\cos(2\theta )$$

where *D_gap_* is the total optical path of the air gap between the two lenses, *D_AB_* is the optical path of the two points A and B, *D_AC_* is the optical path of the two points A and C, *D_CD_* is the optical path of the two points C and D, *n*_1_ is the refractive index of the air, *l* is the vertical distance of the two lenses, and *θ* is the rotation angle. If the rotation angle *θ* is zero, the optical path is minimized. Then:

(4) $$D_{gap}{=n}_{1}l$$

According to Equation (3), we can find the measurement result after the rotation is slightly larger. In Equation (3), considering the rotation angle *θ* is very small, the second term is:

(5) $$n_{1}l\sin^{2}\theta /\cos(2\theta )\approx n_{1}l\sin^{2}\theta =o(\theta )$$

To simplify Equation (3), we ignore high-order small quantities. Thus:

(6) $$D_{gap}{=n}_{1}l/\cos\theta$$

(7) $$\frac{\partial D_{gap}}{\partial \theta }=\frac{n_{1}l\sin\theta }{\cos^{2}\theta }$$

According to Equation (7), when *θ* is zero, the derivative of *D_gap_* is zero. Thus, the total optical path of the air gap reaches a minimum. In summary, no matter if the rotation angle is positive or negative, as long as rotation occurs, the optical path *D_gap_* will become larger than the initial length after this rotation. This will cause the measurement result to be slightly larger and generate measurement error. To improve the accuracy of measurement, the measurement error caused by rotation must be reduced.

#### 3.2.2. Measurement Error Elimination

According to Equation (6), if we obtain the value of the rotation angle through measurement, the measurement error caused by the rotational component can be compensated. In the measurement system reported in this paper, considering the right lens is fixed on the sample rigidly, the rotational angle of the right lens is equal to the rotational angle of the sample.

(8) $$t=\frac{D_{lens}}{n_{2}}=\frac{D_{O_{1}}{}_{O_{2}}+D_{O_{2}}{}_{O_{3}}+D_{O_{3}}E}{n_{2}}=t_{0}/\cos\theta +\frac{n_{1}t_{0}\sin^{2}\theta /\cos(2\theta )}{n_{2}}$$

where *D_lens_* is the optical path of the right lens, *t*_0_ is the original thickness of the right lens, *n*_1_ is the refractive index of the air, *n*_2_ is the refractive index of the right lens, and *θ* is the rotation angle. If the rotation angle *θ* is zero, the thickness of the right lens is $t=t_{0}$.

In Equation (8), considering the rotational angle *θ* is very small, thus the second term is:

(9) $$\frac{n_{1}t_{0}\sin^{2}\theta /\cos(2\theta )}{n_{2}}\approx \frac{n_{1}t_{0}\sin^{2}\theta }{n_{2}}=o(\theta )$$

To simplify Equation (8), we ignore high-order small quantities. Thus:

(10) $$t=t_{0}/\cos\theta$$

Then, according to Equation (10), the rotational angle is:

(11) $$\theta =\arccos(\frac{t}{t_{0}})$$

If the value of *t* is obtained, the rotational angle can be calculated according to Equation (11). In this measurement system, the thickness of the right lens and the air gap can be measured simultaneously using Lenscan 600. Thus, it can guarantee the rotational angle of the right lens, and the distance of the air gap is measured synchronously. When the sample rotates, the rotational angle of the sample can be obtained by comparing the measured thickness of the right lens with the initial thickness. Thus, the measurement error of the rotational component can be eliminated by using Equation (6).

#### 3.2.3. Calculation of CTE

Generally, the variation in the sample’s length during the heating process can be obtained by comparing the optical path of the air gap measured at different temperatures T_1_ and T_2_. To improve the accuracy of calculation, the change in the refractive index of the air and lens with the temperature must be considered. The CTE of the lens should be considered too.

(12) $$\Delta d=\frac{D_{{\mathrm{gap}}_{T2}}-D_{{\mathrm{gap}}_{T1}}}{n_{1_{T2}}}\cos\theta$$

where Δ*d* is the deformation of the sample along the *x*-axis from temperature T_1_ to T_2_, *n*_1_*_T_2__* is the refractive index of the air at temperature T_2_, *D_gapT_*_1_ and *D_gapT_*_2_ are the optical paths of the air gap between the two lenses at different temperatures, and the rotation angle *θ* is the parameter to be measured.

The rotational angle of the sample can be obtained by comparing the measured thickness of the right lens with the initial thickness if the temperature does not change. However, the temperature increases during CTE measurement. The measured thickness of the right lens will include thermal expansion deformation and optical path variation induced by refractive index of the right lens. These factors must be removed from the measurement result. Thus, the pure thickness of the right lens is:

(13) $$t=\frac{D_{lens}}{n_{2}{}_{{}_{T2}}}-\alpha_{lens}t_{0}(T_{2}-T_{1})$$

where *D_lens_* is the optical path of the right lens, *n*_2__*T*_2__ is the refractive index of the right lens at temperature T_2_, and *α_lens_* is the CTE of the right lens. The rotational angle can be calculated according to Equation (11).

Then, combining Equations (11)–(13), the pure deformation of the sample can be obtained. Thus, the thermal expansion coefficient is:

(14) $$\alpha =\frac{\Delta d}{l(T_{2}-T_{1})}$$

## 4. Experiment

In order to verify the effectiveness of the experimental measurement system, we used a truss structure as a reference. It was manufactured by electric discharge machining from a 5 mm thick plate of aluminum. The properties of the glass and air are provided in Table 1. The refractive index of the air at different temperatures was calculated according to the equation of Rüeger [22]. The effects of the translational component and rotational component on the measurement result were verified by the experiment. Then, the CTE along the length of the aluminum sample was measured to verify the validity of this experimental system.

### 4.1. Translational Experiment

**Figure 7:**
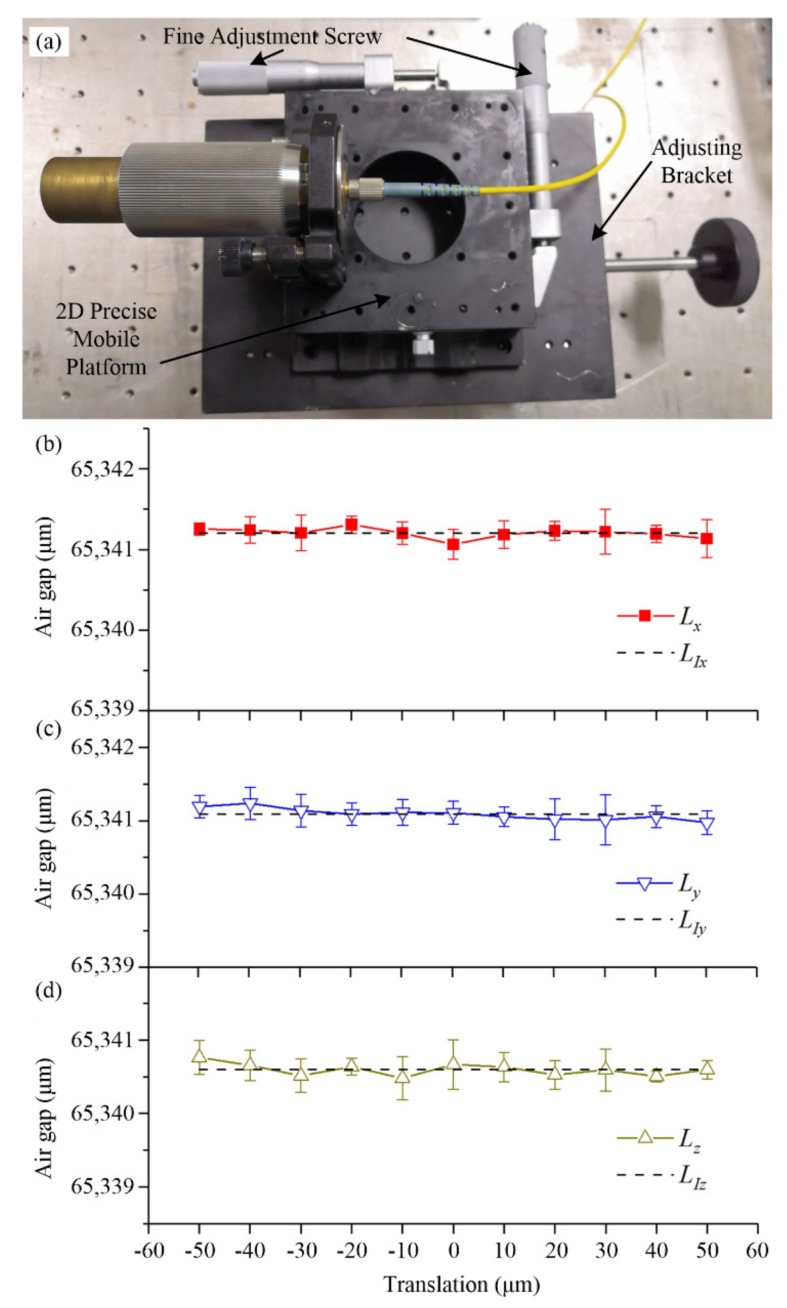
Measurement results after artificial additional translation. (**a**) The translation adjustment device. (**b**) Measurement results after *x*-axis translation. “*L_x_*” is the length of the air gap after *x*-axis translation. (**c**) Measurement results after *y*-axis translation. “*L_y_*” is the length of the air gap after *y*-axis translation. (**d**) Measurement results after *z*-axis translation. “*L_z_*” is the length of the air gap after *z*-axis translation. The dashed lines are the initial length of the air gap before translation.

The *x* direction displacement of the laser was adjusted using the adjusting bracket. The *y* and *z* direction displacements of the laser were adjusted by a 2D precision mobile platform as shown in Figure 7a. The position of the laser was detected by a micrometer (Shahe5313-02, Zhejiang, China) with an accuracy of ±2 μm. According to the measurement steps described in Section 2.3, the temperature was kept at room temperature (i.e., 20 °C); the laser was moved respectively along the *x*, *y*, and *z* directions from −50–50 μm; the interval was 10 μm; and each position was measured five times. The effects of the translational component on the length of the air gap are shown in Figure 7, and the measured lengths of the air gap after translating in the *x*, *y*, and *z* directions are provided in Figure 7b–d, respectively. “*L_x_*” is the length of the air gap after *x*-axis translation. “*L_y_*” is the length of the air gap after *y*-axis translation. “*L_z_*” is the length of the air gap after *z*-axis translation. “*L_Ix_*”, “*L_Iy_*”, and “*L_Iz_*” are the initial lengths of the air gap represented by the dashed lines in Figure 7b–d, respectively. According to the results, the lengths of the air gap fluctuate around the mean length. The amount of fluctuation was less than the accuracy of the measurement (±1 μm). Thus, the lengths of the air gap had no obvious trend with translation. This proves that translation had little effect on the length measurement. This is consistent with the previous analysis.

### 4.2. Rotational Experiment

**Figure 8:**
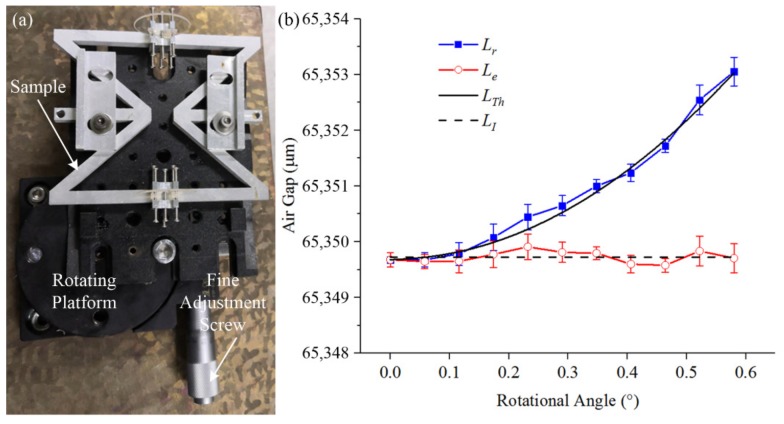
Measurement results after artificial additional rotation. (**a**) The experimental device of the sample rotation. (**b**) Measurement results after rotation. “*L_r_*” is the measurement length of the air gap after rotation. “*L_Th_*” is the theoretical length of the air gap after rotation. “*L_e_*” is length of the air gap after elimination of the measurement error of rotation. The dashed line is the initial length of the air gap before rotation.

The rotation of the sample was realized by a rotation platform (KSP-656M, Guangdong, China) with an accuracy of ±52.2″ as shown in Figure 8a. The temperature was kept at room temperature (i.e., 20 °C); the fine adjustment screw was adjusted to rotate the sample around the *y*-axis from 0 to 0.6°; the interval was 0.058°; each position was measured five times. Figure 8b shows the effect of the rotation on the sample length measurement at 20 °C. “*L_r_*” is the measurement length of the air gap after rotation. “*L_Th_*” is the theoretical length of the air gap after rotation. “*L_e_*” is length of the air gap after eliminating the measurement error of rotation. The dashed line “*L_I_*” is the initial length of the air gap before rotation. According to the measurement result, the measurement length of the air gap increased when the rotation angle increased. This proves that the measurement length of the air gap after rotation was larger than the actual length. The rotational component generates extra measurement error. It will reduce the measurement accuracy of the thermal expansion coefficient. In Figure 8b, the red line provides the length of the air gap after eliminating the measurement error of rotation. This proves that the elimination was effective in reducing the measurement errors caused by rotation. Therefore, in order to improve the measurement accuracy, it is necessary to take measures to eliminate the measurement errors caused by rotation.

### 4.3. Measurement of the CTE of the Sample

According to the measurement steps described in Section 2.3, with interval of 20 °C, the sample was heated to 120 °C. The length of the air gap was measured five times at each temperature. The sample was heat-treated to reduce the residual stress. This reduces the warping deformation of the sample during measurement.

**Figure 9:**
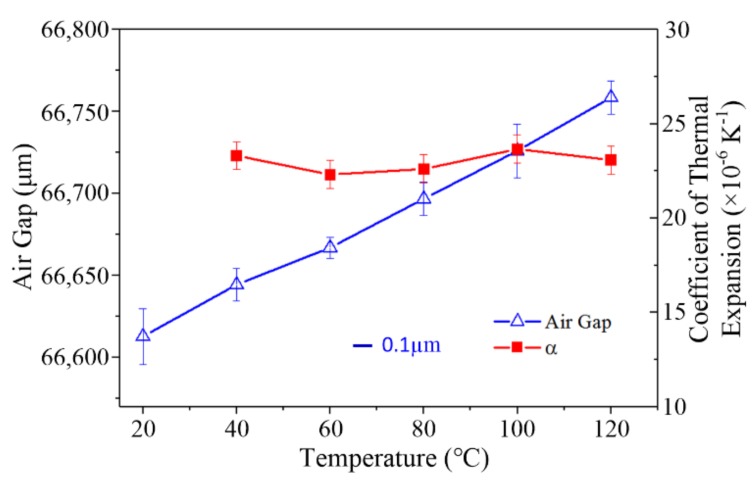
CTE measurement of the aluminum truss structure. The air gap is the distance between the two lenses fixed on the sample. It increased with an increase in the temperature. The blue scale bar represents 0.1 µm in length of the measurement error of the air gap. “α” is the CTE of the sample.

As shown in Figure 9, The “Air Gap” is the distance between the two lenses fixed on the sample. The length of the air gap between the two lenses increased with the increase in temperature. The blue scale bar represents 0.1 µm in length of the error bar of the air gap. “α” is the equivalent CTE of the sample. The average CTE of the sample was 22.99 ± 0.54 × 10^−6^ K^−1^. Compared with the recommended value (23.1 × 10^−6^ K^−1^) in the existing literature [24], this proves that this experimental system is valid and can be used for measuring the thermal expansion of truss structures.

## 5. Discussion

Generally, the measurement accuracy of the thermal expansion coefficient depends on the accuracy of the temperature control and length measurement. The accuracy of the temperature control can be improved by advanced temperature control technology and high precision temperature sensor. In addition, the accuracy of the length measurement by laser interferometry has reached the nanometer scale. Thus, the measurement error will become the key factor to improving measurement precision. Vibration isolation is a good error control method in most cases. However, with temperature changes, it will bring many uncertain micro-displacements which cannot be suppressed by vibration isolation. This method can effectively remove the measurement error caused by the micro-displacements. It will reduce the isolation requirements for the environment and enhance the anti-interference ability. In addition, with the development of modern grating processing and high precision rail technology, interferometers with higher precision will be manufactured. The measurement accuracy of this method can be further improved.

This method has the potential to eliminate the measurement error caused by micro-displacement, and its effectiveness was verified by the experiment in Section 4. However, limited by the length measurement accuracy of the Lenscan 600, it cannot be used to eliminate the rotation error in actual measurements. In this presented measurement system, the rotational angle of the sample was obtained by detecting the thickness change of the right lens. Considering that the measurement accuracy of the Lenscan 600 is ±1 μm, the minimum detection angle of the lens with a thickness of 0.75 mm is 3°. This angle is beyond the angular tolerance of Lenscan 600 and far more than the actual rotational angle. One possible approach to decrease the minimum detection angle is to increase the thickness of the right lens. If the thickness of the right lens is 10 mm, the minimum detection angle is 0.81°. If the thickness of the lens is 656 mm, the minimum angle that can be measured is 0.1°. This method requires a large thickness of lens in order to achieve a sufficient angular detection accuracy. Hence, limited to the length measurement accuracy in the experimental system, this method is difficult to apply in actual measurement.

With the development of high-precision measuring instruments, especially with the accuracy of interferometry reaching 1 nm, this method could achieve sufficient angular detection accuracy. Assuming the measurement accuracy of the distance measuring system is 1 nm, if the thickness of the lens is 1 mm, the minimum detection angle is 0.08°. If the thickness of the lens is 20 mm, the minimum angle that can be measured is 1.5′. Then, the angular detection accuracy would be sufficient. The measurement error caused by rotation can be eliminated by this method. This method can effectively improve the accuracy of thermal expansion measurement. This is meaningful for the design and fabrication of zero thermal expansion lattice. Furthermore, it can be determined from theoretical analysis that rotational error can be eliminated as long as the rotational angle is obtained. There are several other approaches that can also achieve high-precision angle measurement such as high-precision electronic autocollimator and lens surface measurement. With these methods, we expect that the measurement error can be eliminated accurately. This method could greatly improve the anti-interference ability and accuracy by eliminating the measurement error.

The lattices with tailorable coefficients of thermal expansion are realized using two or three kinds of materials to form complex truss structures. Some of the structures are symmetric and the thermal expansion of the structures are axial elongation or shrinkage, without a rotational component. Considering a more general situation where the truss structures are asymmetric, these would have different stiffnesses in each direction. Then, the thermal expansion of the designed structures would have an axial component and a rotational component. Even though this method can eliminate the measurement error, it still has some restrictions. It cannot be used to measure the complex thermal deformation when there is an axial component and a rotational component. This method tests the CTE by measuring the length variation of the lattice. It can only measure the change in distance between two points along the optical axis without rotation. If thermal deformation of the dual-material lattice contains both of the two components, the rotational component of the CTE will mix with the rotational error. This will lead to a wrong result. Thus, this method cannot be used to measure the CTE of the axial component and the rotational component simultaneously. One possible solution is to measure the CTE of each component alone using this method and then to obtain the total CTE though calculation.

## 6. Conclusions

In this paper, we reported on a robust interferometric method that can eliminate the measurement error induced by micro-displacement. This method can greatly enhance the anti-interference ability and accuracy by eliminating the measurement error. This method is especially useful for CTE measurement of non-standard samples like dual-material truss structures with high precision requirements. The theoretical analysis indicated that translation did not affect the measurement results and that rotation made the measurement results lager. The robust experimental system was designed and built using two plane lenses and a distance measuring interferometer. The experimental results of the aluminum truss structure proved that this method can avoid the translation error and has the potential to eliminate the measurement error caused by rotation. The CTE measurement results of the sample successfully demonstrated the validity of the measurement system.

## Figures and Tables

**Table 1:** Properties for the glass [23] and air [22].

Material	Refractive Index	Temperature Coefficient of the Refractive Index (×10^−6^ K^−1^)	Coefficient of Thermal Expansion α (×10^−6^ K^−1^)
Glass N-BK7	1.5035829	2.4	8.3
Air	1.000271	-	-
